# Parallel Patterns of Increased Virulence in a Recently Emerged Wildlife Pathogen

**DOI:** 10.1371/journal.pbio.1001570

**Published:** 2013-05-28

**Authors:** Dana M. Hawley, Erik E. Osnas, Andrew P. Dobson, Wesley M. Hochachka, David H. Ley, André A. Dhondt

**Affiliations:** 1 Department of Biological Sciences, Virginia Tech, Blacksburg, Virginia, United States of America; 2 Department of Ecology and Evolutionary Biology, Princeton University, Princeton, New Jersey, United States of America; 3 Cornell Lab of Ornithology, Cornell University, Ithaca, New York, United States of America; 4 Department of Population Health and Pathobiology, College of Veterinary Medicine, North Carolina State University, Raleigh, North Carolina, United States of America; The Pennsylvania State University, United States of America

## Abstract

The evolution of higher virulence during disease emergence has been predicted by theoretical models, but empirical studies of short-term virulence evolution following pathogen emergence remain rare. Here we examine patterns of short-term virulence evolution using archived isolates of the bacterium *Mycoplasma gallisepticum* collected during sequential emergence events in two geographically distinct populations of the host, the North American house finch (*Haemorhous* [formerly *Carpodacus*] *mexicanus*). We present results from two complementary experiments, one that examines the trend in pathogen virulence in eastern North American isolates over the course of the eastern epidemic (1994–2008), and the other a parallel experiment on Pacific coast isolates of the pathogen collected after *M. gallisepticum* established itself in western North American house finch populations (2006–2010). Consistent with theoretical expectations regarding short-term or dynamic evolution of virulence, we show rapid increases in pathogen virulence on both coasts following the pathogen's establishment in each host population. We also find evidence for positive genetic covariation between virulence and pathogen load, a proxy for transmission potential, among isolates of *M. gallisepticum*. As predicted by theory, indirect selection for increased transmission likely drove the evolutionary increase in virulence in both geographic locations. Our results provide one of the first empirical examples of rapid changes in virulence following pathogen emergence, and both the detected pattern and mechanism of positive genetic covariation between virulence and pathogen load are consistent with theoretical expectations. Our study provides unique empirical insight into the dynamics of short-term virulence evolution that are likely to operate in other emerging pathogens of wildlife and humans.

## Abstract 2

A bacterial pathogen of wild songbirds evolved higher virulence following its emergence in two separate regions of the host range.

## Author Summary

A long-standing paradox in the study of infectious diseases is why pathogens evolve to cause harm to the very hosts they depend on to survive and reproduce. Research over several decades suggests that this harm, or virulence, is an inevitable by-product of the pathogen replication needed to maximize the chance that a given pathogen will be transmitted to another host. Here we demonstrate that a recently emerged bacterial pathogen of a North American songbird species has gradually become more virulent during each of two emergence events in different regions of the host range. This evolution of higher virulence appears to have been driven by selection for high rates of pathogen replication, because bacterial isolates that are more virulent in finches also attain the highest loads in infected host tissues. Overall, our results indicate that emerging pathogens can evolve to become more virulent in their hosts, at least in the short term, when an increase in the pathogen's ability to replicate is linked with higher virulence. Our findings have important implications for understanding and predicting the severity of disease caused by emerging pathogens in wildlife, domestic animals, and humans.

## Introduction

The extensive variation in the amount of harm that pathogens cause their hosts has intrigued biologists for centuries and has generated several decades of theoretical work to explain the evolution of pathogen virulence (e.g., [1]–[4]). Despite this broad and long-standing interest, empirical studies of virulence evolution in non-laboratory systems remain rare (but see [5]–[10]). Furthermore, very few theoretical studies have addressed the short-term dynamics of virulence evolution, which are likely to differ from expected long-term or evolutionarily stable outcomes [4],[11]–[15]. As new pathogens continue to emerge there is a growing need to understand the short-term dynamics of virulence evolution and the ecological and anthropogenic mechanisms that may influence these dynamics [16],[17].

The seminal work of theoretical biologists in the 1980s–1990s on the evolution of virulence is regarded as one of the most important recent developments in population and evolutionary biology (e.g., [1],[2],[18],[19]). This body of theory assumes a virulence “trade-off” and disproved the long-standing hypothesis that evolutionary interactions of parasites and pathogens would necessarily evolve to the lowest possible level of virulence. The trade-off theory's key assumption is that virulence is intimately coupled to transmission because of the dependence of both traits on host exploitation. Thus, the within-host rate of pathogen replication necessary for transmission will also directly (or indirectly) reduce host fitness (i.e., cause the pathology associated with virulence). If increased virulence and transmission to a new host are both associated with higher pathogen load, under certain conditions virulence is expected to evolve toward an intermediate value that is evolutionarily stable [1],[2],[4],[11],[18]–[21].

The assumptions and predictions of the trade-off model have been tested empirically in a number of systems (reviewed in [22]). However, opportunities to investigate the evolution of virulence during the disease emergence process, when changes in virulence represent short-term rather than equilibrium dynamics, have been rare [5],[23],[24]. Arguably the best study of the evolution of virulence following pathogen emergence is of *Myxoma* virus in rabbits, where the evolution of lower and then intermediate pathogen virulence was documented following the initial introduction of a highly virulent strain into naïve rabbit populations in both Australia and Europe [5],[25],[26]. Although the rabbit–*Myxoma* system suggests that changes in virulence can occur rapidly following pathogen emergence, the initial emergence of myxomatosis resulted from an artificial introduction of a highly virulent strain as a means of rabbit population control. The extent and direction of short-term virulence evolution remain unknown in emerging disease systems more broadly [27]. Theoretical work on nonequilibrium virulence evolution during disease emergence [12],[13] predicts that, if transmission and virulence positively covary, selection will favor increasing virulence early in an epidemic, while susceptible hosts are common. Furthermore, positive genetic correlations between transmission and virulence among pathogen strains can result during disease emergence from demographic processes even in the absence of the constraints that underlie the trade-off model [12], and these correlations can alone result in increasing virulence via indirect selection on transmission.

One of the best-studied emerging wildlife disease systems is the infection of house finches (*Haemorhous* [formerly *Carpodacus*] *mexicanus*) by the bacterial pathogen *Mycoplasma gallisepticum* (MG) [28]–[31] that is spread by direct contact or short-term indirect contact on bird feeders [32]. A novel strain of MG emerged, presumably from poultry, and spread within introduced populations of eastern North American house finches in the mid-1990s [29],[33]. At the time of MG emergence, eastern North American house finch populations were still expanding westward from an initial small colony of individuals introduced on the east coast from the pet trade in the 1940s [34]. After rapidly spreading through introduced host populations, MG then spread slowly further westward [35], emerging in the now-contiguous native, western range of house finches in the early 2000s [36]–[38]. Westward spread of MG was slowed by relatively low house finch densities across the Great Plains [39] and the historically separate nature of eastern and western house finch populations that only recently became contiguous [39]. Because western house finches are essentially nonmigratory [40] and show significant population genetic structure from eastern populations at multiple loci [41],[42], admixture between eastern and western house finch populations is likely minimal.

Infection with MG results in severe conjunctival inflammation in house finches [31], ultimately reducing overwinter survival rates for affected individuals [43]. Notably, the emergence of MG caused up to 60% declines in previously expanding eastern North American house finch populations [44]. Although MG apparently emerged as a single strain in house finches [33],[45], both genotypic [33],[46]–[48] and phenotypic [49] differentiation among MG isolates from songbirds have now been documented. In particular, the virulence of the earliest detected MG isolate on the west coast of the United States (CA2006) is significantly lower than that of the initially emerged isolate in the eastern United States (VA1994) [49]. Because a phylogeny of archived isolates indicates that a single lineage of MG derived from an eastern isolate colonized the west coast of North America (W. M. Hochachka, A. A. Dhondt, A. P. Dobson, D. M. Hawley, D. H. Ley, et al., unpublished data), we can consider the emergence of MG in eastern and western house finches as replicate events in order to examine whether phenotypic virulence systematically changed following the initial emergence of MG in geographically separate host populations.

Here we show that virulence increased following the sequential emergence of MG in eastern and western North American house finches, a pattern consistent with theoretical predictions for short-term virulence evolution [12],[13]. Also consistent with theoretical assumptions, we describe positive correlations between indices of transmission and virulence among isolates of MG circulating in North American house finches. We present results from two complementary experiments, one that examines the trend in pathogen virulence in eastern North American MG isolates collected from a limited geographic region over the course of the epidemic (1994–2008), and the other a parallel experiment using Pacific coast isolates of the pathogen collected after MG established itself in western house finch populations (2006–2010). Our experiments provide important insights into the patterns and mechanisms of virulence evolution during disease emergence that are likely to also apply to emerging pathogens less amenable to detailed experimental study.

## Results

### Host and Experiment Effects

Our experimental design minimized potential contributions of host–pathogen coevolution by assaying virulence using finches from a different geographic area than that of the bacterial isolates to which they were exposed (i.e., eastern MG isolates were assayed in western finches, and western isolates in eastern finches; Table 1). Our prior work found no evidence of differences in host response to two MG isolates between house finches from eastern (New York) and western (California) North America [49]. Nevertheless, because we used hosts from two geographic areas in our two experiments, we tested for potential differences due to host origin by infecting finches of both geographic origin with the same reference isolate—the original index MG isolate (VA1994) collected in June 1994 from a Virginian house finch with conjunctivitis [28]—as part of experiment 2 (Table 1). We then examined the effect of experiment and host origin on virulence for VA1994 because this isolate was the only one used in both experiments and inoculated into hosts of both geographic origin. Because we found significant host and experimental effects for VA1994 (Figure S1), our quantitative inferences are restricted to within-experiment and within-host-population comparisons only. Qualitative between-experiment inferences are made only with respect to the reference isolate (VA1994).

**Table 1:** The study design for experiments 1 and 2, including isolates and dilutions used, the number of hosts inoculated, and host origin.

Experiment	MG Isolate	Concentration (CCU/ml)	Dilution Factor	*N* Hosts Inoculated	Host Origin
1	Reference isolate: VA1994 (7994-1-7P)	2.24×10^7^	1∶1	10	AZ
1	NC1995 (13295-2-5P)	8.56×10^7^	1∶3.82	11	AZ
1	NC1996 (1596-4-3P)	3.04×10^8^	1∶13.57	11	AZ
1	NC2006 (2006.080-5-4P)	3.04×10^8^	1∶13.57	11	AZ
1	NC2008 (2008.031-4-3P)	1.87×10^8^	1∶8.35	11	AZ
1	Negative control (FMS)	n/a	n/a	10	AZ
2	Reference isolate: VA1994 (7994-1-7P)	2.24×10^7^	1∶7.37	15	AL (*n* = 7)/AZ (*n* = 8)
2	CA2006 (2006.052-5-4P)	3.04×10^6^	1∶1	8	AL
2	CA2008 (2008.028-2-3P)	7.44×10^6^	1∶2.45	10	AL
2	CA2009 (2009.061-1-3P)	1.87×10^7^	1∶6.15	10	AL
2	CA2010 (2010.003-1-3P)	4.60×10^7^	1∶15.1	10	AL
2	Negative control (FMS)	n/a	n/a	6	AL (*n* = 5)/AZ (*n* = 1)
2	Behavioral controls (FMS)	n/a	n/a	13	AL

AL, Alabama; AZ, Arizona; CCU, color changing units; n/a, not applicable.

### Temporal Patterns of Virulence

**Figure 1:**
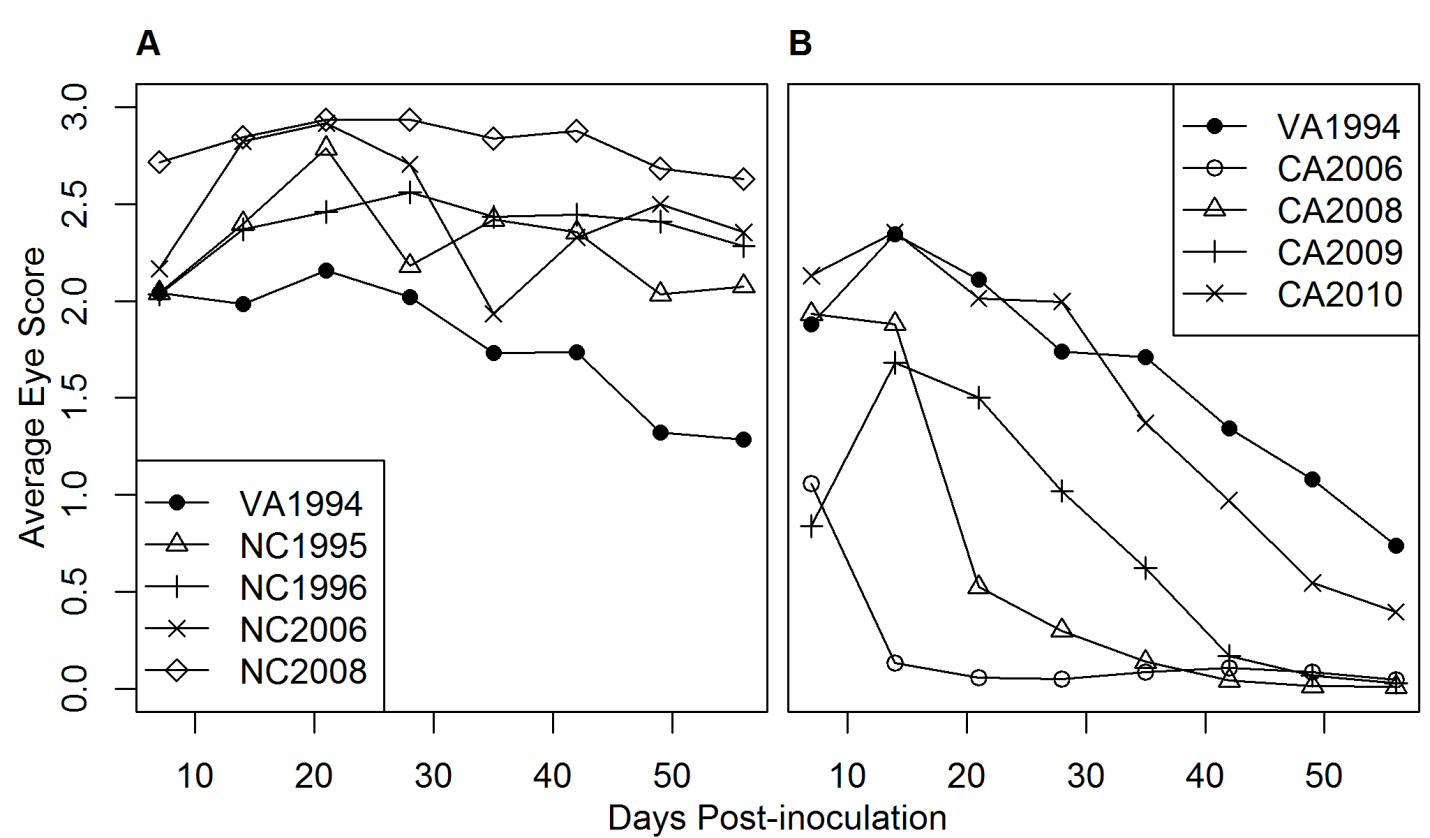
Average eye score of house finches inoculated with *M. gallisepticum* isolates. Isolates were eastern (A) (experiment 1) or western (B) (experiment 2) in origin, and means are presented for each observation day PI.

**Figure 2:**
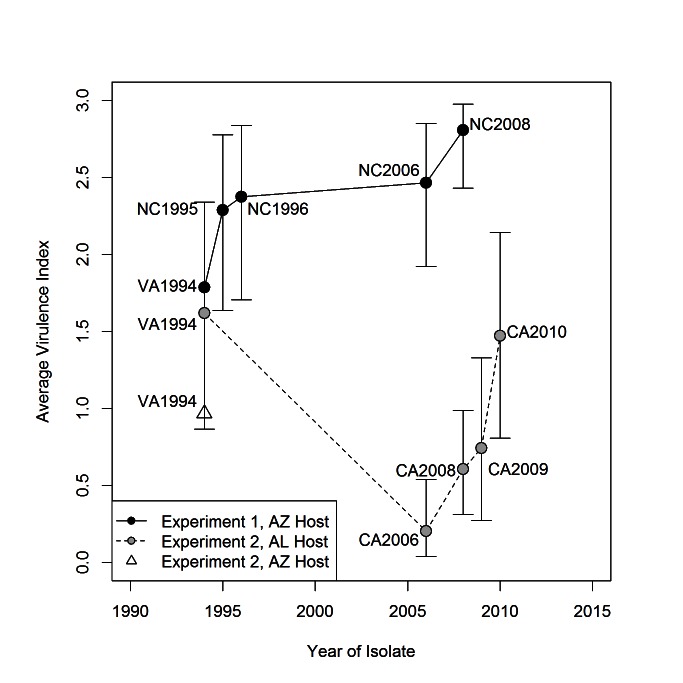
Average virulence index for each isolate of *M. gallisepticum* by year of isolation. The average virulence index was calculated as the average response value over the eight sampled days PI (plotted separately in Figure 1). Each data point is labeled with the corresponding isolate (Table 1), and points connected by lines are from the same experiment and host population (AZ = Arizona, AL = Alabama). Bars represent 95% Bayesian credible intervals.

On both the east and west coasts of North America, MG isolates that were collected in successive years after initial discovery in that region were increasingly more virulent as measured by eye score (Figures 1 and 2). Consistent with prior results [49], the earliest detected isolate on the west coast (CA2006) was substantially less virulent than the east coast reference isolate (VA1994; Figure 2). However, subsequent isolates circulating in the west increased in virulence in successive years after discovery in ways that closely resembled the increase in virulence of successive isolates that circulated on the east coast. After only four years, the most recently collected west coast isolate (CA2010) produced an eye score response similar to that of the original east coast reference isolate (VA1994; Figure 2). Although we cannot make robust quantitative comparisons, the rate of increase in virulence, as measured by eye score, was approximately 3.5 times faster on the west coast.

### Among-Isolate Covariation in Virulence and Pathogen Load

**Figure 3:**
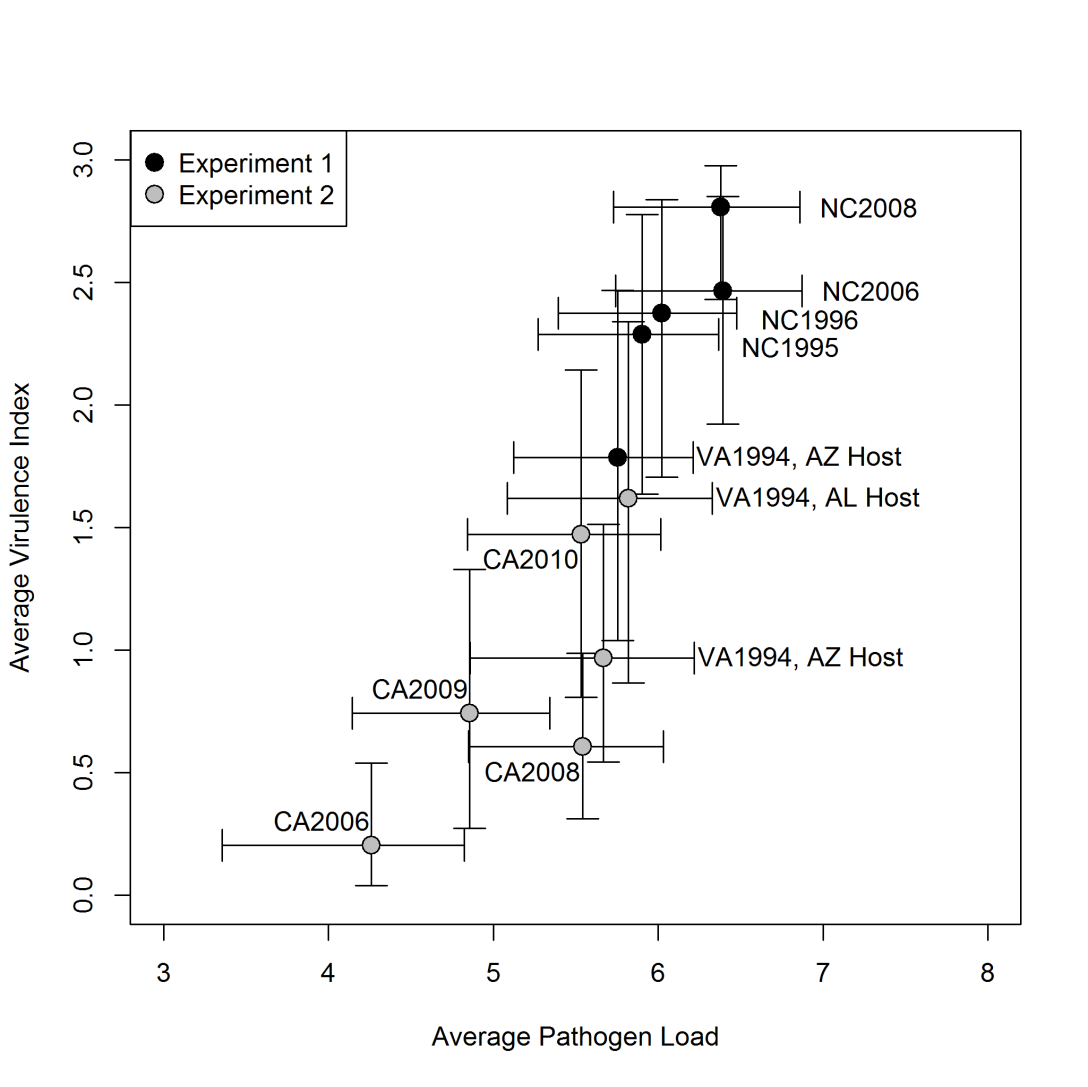
Average pathogen load (log_10_ scale) and average virulence index positively covary among eastern and western *M. gallisepticum* isolates. Each data point is labeled with the corresponding isolate (filled circles for eastern isolates and open circles for western isolates), and the reference isolate (VA1994) is plotted separately for each experiment–host combination. Bars represent 95% Bayesian credible intervals.

There was a strong positive relationship between average virulence index (the mean pathogen component of virulence over the sampling period; Equation 5, Methods S1) and average pathogen load (the mean pathogen load for a given isolate over the sampling period; Equation 5, Methods S1) among the examined isolates (Figure 3). The posterior mean correlation between our indices of virulence and pathogen load was 0.65 (95% credible interval, 0.42, 0.84) when experiment effects were not removed, and 0.39 (95% credible interval, 0.02, 0.70) within an experiment after controlling for differences in means between the two experiments (see Materials and Methods).

### Geographic Patterns of Virulence

**Figure 4:**
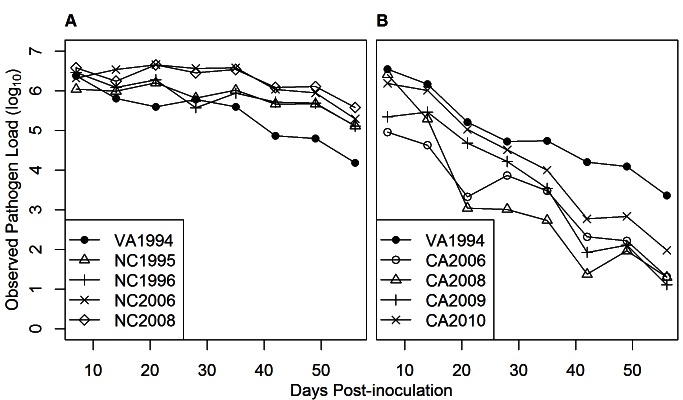
Observed pathogen load (log_10_ scale) in the conjunctiva of house finches inoculated with *M. gallisepticum* isolates. Isolates were eastern (A) (experiment 1) or western (B) (experiment 2) in origin, and means are presented for each observation day PI.

The general among-isolate pattern of covariation (Figure 3) that we detected was underlain by qualitatively different within-host trajectories of virulence and pathogen load over the course of infection between the east and west coast isolates. More specifically, infection by west coast isolates led to lower virulence and pathogen loads (relative to the reference isolate) later in infection compared to infection by east coast isolates. For the east coast, all isolates maintained a relatively high eye score until day 56 post-inoculation (PI) compared to the reference isolate (VA1994; Figure 1A). For west coast isolates, eye scores were initially similar to or lower than those for the reference isolate (VA1994) and then tended to drop more quickly through time (Figure 1B). For pathogen load, results were qualitatively similar: east coast isolates maintained high observed pathogen load relative to the reference isolate until day 56 PI (Figure 4A), whereas the pathogen load of west coast isolates fell rapidly after day 7 PI in comparison to the reference isolate (Figure 4B). When the two components of observed pathogen load (probability of observing a non-zero pathogen load (Figure S3), and expected pathogen load given a non-zero result (Figure S2; see Methods S1) were examined separately, a qualitatively similar pattern was found for expected and observed pathogen load. However, expected pathogen load decreased more slowly for the west coast isolates (Figure S2B) than did observed pathogen load (Figure 4B). The probability of observing a non-zero pathogen load was high for east coast isolates across all days PI relative to the reference isolate (Figure S3A). In contrast, these probabilities decreased rapidly with day PI for west coast isolates compared to the reference isolate (Figure S3B).

## Discussion

Consistent with predictions from current theory on short-term virulence evolution, we documented rapid increases in virulence of MG not only during its initial emergence as a pathogen of house finches in eastern North America, but also during subsequent emergence of the pathogen in a second and geographically separate population of house finches in western North America (Figure 2). This second increase came after a transient decrease in virulence [37],[49] seemingly associated with dispersal of the pathogen to the Pacific coast. Furthermore, these evolutionary changes appear to have been driven by positive genetic covariation between virulence and transmission among isolates (Figure 3).

Because phylogenetic analyses indicate that western isolates represent a monophyletic clade derived from eastern isolates (W. M. Hochachka, A. A. Dhondt, A. P. Dobson, D. M. Hawley, D. H. Ley, et al., unpublished data), western isolates may have been genetically predisposed to evolve higher virulence because of the selection occurring in eastern North America. However, the western clade of MG apparently also underwent selection for decreasing virulence ([49] and this study). Theory predicts that spatial structure associated with low dispersal and movement can favor low pathogen virulence via the competition–persistence trade-off (reviewed in [50]), and several experimental studies support this theoretical prediction ([51]–[53], but see [54]). Eastern and western house finch populations only recently became contiguous [40] and are at lowest density across the Great Plains [39], which likely limited the ability of MG to disperse westward and did not provide the large numbers of susceptible hosts needed for short-term evolution of higher virulence. Furthermore, if long-distance movement of infected house finches decreases with higher virulence, the low virulence of the earliest collected isolate in western North America (CA2006) may have resulted from higher westward pathogen dispersal of low-virulence isolates. House finches with mycoplasmal conjunctivitis decrease their rate of movement both in the wild [55],[56] and in captivity [31], providing support for a movement cost to virulence in this system. Our results are therefore consistent with low host density in the Great Plains and movement costs of virulence underlying the decrease in virulence observed as MG spread and colonized the west coast of North America.

The pattern, comparable to that of the east coast, of increasing virulence once MG established itself in naïve west coast populations of house finches, initiating a second short-term virulence cycle, strongly suggests that similar selective pressures operated on virulence evolution during these sequential emergence events. Specifically, the positive covariation we detected between pathogen load and virulence among isolates on both coasts (Figure 3) suggests that the evolution of higher virulence was an indirect result of selection for increased pathogen load and transmission potential in populations with large numbers of susceptible hosts. If there are simple linear relationships between virulence and average eye score and between transmission and pathogen load in our system, the positive correlation we detected between average virulence index and average pathogen load (Figure 3) shows support for one of the central assumptions of the trade-off theory of virulence evolution [57]. However, this support needs to be tempered by our lack of knowledge about the strength and shape of the relationship between eye scores and rates of disease-induced mortality in the wild, and between average pathogen load and transmission probability. Although we did not measure transmission in this study, unpublished work on three MG isolates that span the detected range of virulence (CA2006, VA1994, and NC2006; Figure 2) found a positive relationship between average pathogen load and among-host rates of transmission (Figure S4; Methods S2). Therefore, as has been shown in other systems [58]–[60], average pathogen load is likely a reasonable proxy of transmission potential for a given MG isolate under certain ecological conditions. However, further work is needed on larger numbers of isolates in order to determine whether the relationship between pathogen load and transmission is linear or saturating in this system, as the shape of this relationship is expected to significantly influence virulence evolution in the long term.

We detected effects of both host origin and experiment (1 versus 2) in our study (Figure S1; host differences explored in detail in [61]). Because of these effects, and recent work by Bonneaud and colleagues that detected differences in pathogen load [62] and immune responses [62],[63] between Arizona and Alabama house finches in response to MG infection, we limited our qualitative interpretations about virulence evolution to within rather than between experiments. Although we were able to statistically estimate and control for host effects in response to the reference isolate (VA1994), there may be isolate-by-host interaction effects on virulence that our experimental design was unable to test for. Potential host-by-isolate interactions would not alter our interpretation of virulence trajectories on either side of North America, but could influence the shape of the relationship between pathogen load and virulence among isolates (Figure 3). However, because the correlation between pathogen load and virulence was still positive when calculated only within an experiment, the covariation between pathogen load and virulence necessary for short-term virulence evolution is present both within and between the two experiments in our study.

### Potential Selective Mechanisms Contributing to Increased Virulence

The strong positive covariation we detected between pathogen load and virulence is consistent with a simple selection process whereby selection for increased transmission results indirectly in higher virulence because of the dependence of both traits on pathogen load. Although there are several potential mechanisms that may have contributed to this process, we hypothesize that two mechanisms may have been particularly important in this system.

First, within-host mechanisms such as host immunity, which has been implicated in driving pathogen virulence evolution in both experimental and natural systems [25],[64],[65], may have contributed to the increases in virulence of MG observed on each coast. The role of host immunity in driving pathogen virulence evolution is particularly important when the immune system's ability to reduce pathogen growth, due to immunity from prior exposure or vaccination, is imperfect [16],[66]. If more virulent isolates of MG (also known as “virulence mutants”; [67]) are better able to reproduce in partially immune house finches, then an isolate with optimal virulence in a partially immune host will express higher than optimal virulence when measured in naïve hosts, as we have used in this study. This mechanism has been shown in Marek's disease virus strains in vaccinated chickens [68]. Under this scenario, the increase in the number of partially immune hosts following MG's emergence on each coast may have selected for increasingly virulent isolates. The conditions are present in our system for partial immunity to contribute to virulence evolution: first, house finches recover from MG infection in the wild [43] and in captivity [31], and individuals that were previously exposed are partially immune upon re-exposure [69]; second, rates of exposure to MG are as high as 50% in some regions [37],[70],[71], providing a strong selective pressure on MG to overcome the partial host immune response.

The second mechanism that may have been particularly important in this system involves anthropogenic features such as bird feeders that may have also contributed to the detected increases in virulence of MG on both coasts. During the nonbreeding season, house finches in most of North America feed extensively at bird feeders, where the majority of MG transmission occurs [30]. Feeders are fomites of MG [32], and the amount of time that susceptible individuals spend on bird feeders predicts the likelihood of transmission in an experimental system (S. L. States, W. M. Hochachka, and A. A. Dhondt, unpublished data). Furthermore, house finches with clinical signs of MG spend significantly more time on bird feeders than healthy conspecifics [55], thus potentially increasing localized transmission. In this system, increasing virulence may therefore be associated with higher rates of local transmission on bird feeders, contributing to positive among-isolate covariation between virulence and transmission potential. It remains unknown whether that relationship would become saturating at high virulence levels, thus ameliorating the transmission advantage of additional increases in virulence.

### Conclusions

Understanding the evolution of virulence is central to a deeper understanding of parasite–host coevolution and, more specifically, to the management of infectious diseases [72], including vaccination strategies [16],[67]. We have demonstrated parallel patterns of virulence evolution in the same pathogen–host system on two sides of a continent, whereby the pathogen rapidly evolved increasing levels of virulence over the first decade of its interaction with each population of the host species. Both the detected evolutionary pattern and the mechanism are consistent with current theory on short-term virulence evolution. Our study therefore provides one of the first empirical examples of short-term virulence evolution in an emerging pathogen. It will be critical to assay a longer time series of isolates from each location in order to determine whether the virulence of MG converges on an evolutionarily stable value and whether that value is similar across localities. We suspect that similar patterns of changing virulence will be detected in other emerging pathogens of wildlife, domestic animals, and humans whenever positive genetic covariation exists between virulence and transmission.

## Materials and Methods

### Ethics Statement

All procedures for animal care and use were conducted in accordance with national guidelines and were approved by Virginia Tech's Institutional Animal Care and Use Committee.

### Experimental Design

Each of our two experiments used house finches from a different population, such that the finches used in a given experiment came from a different geographic area than the bacterial isolates to which they were exposed (Table 1). This design minimizes the potential for confounded effects due to host–pathogen coevolution that may have resulted from the population genetic structure between eastern and western North American house finches previously documented at both neutral [41],[42] and functional [73] genetic markers. First, in experiment 1, in order to examine how the virulence of MG changed following its initial emergence in eastern North American house finches, we experimentally infected house finches from a single population origin in western North America (Arizona) with MG isolates collected from a limited eastern geographic region (Virginia and North Carolina) throughout the course of the eastern North American MG epidemic (1994–2008). Experiment 2 used an identical approach in order to test how the virulence of MG changed following the establishment of the pathogen on the Pacific coast. Again, we selected isolates from a limited western geographic region (California) collected in 2006–2010 following the initial establishment of MG in that region, and assayed these isolates in eastern North American house finches from Alabama. We tested for potential differences in virulence due to host origin and experiment by infecting a small number of finches from Arizona with the reference isolate (VA1994) in experiment 2 (Table 1).

### Bird Capture and Housing

In 2010, 66 house finches from Maricopa County, Arizona, were captured with cage traps (permits from the state of Arizona [SP573456], the US Fish and Wildlife Service [MB158404-1], and the US Geological Survey Bird Banding Laboratory [23513]) and shipped via commercial air carrier to Virginia Tech in February 2010. An additional 64 individuals were captured in Auburn, Alabama, in September 2010 (state of Alabama permit 5436) and transported to Virginia Tech via state vehicle. All house finches were held in individual cages at constant day length (12 h light∶ 12 h dark) and temperature and fed ad libitum pelleted diet (Daily Maintenance Diet, Roudybush) throughout the experiment. All individuals were seronegative for MG at capture.

### Pathogen Inoculation

#### Experiment 1—eastern isolates

All stocks of MG inocula were grown in Frey's broth medium with 15% (volume/volume) swine serum (FMS) [74] and stored at −70°C until immediately prior to use. Upon thawing, inocula were diluted with FMS to approximate the dose (2.24×10^7^ color changing units/ml) of the reference isolate VA1994 (Table 1). On 25 March 2010, *n* = 64 house finches from Arizona were inoculated bilaterally in the palpebral conjunctiva with 0.05 ml of FMS alone (*n* = 10 controls) or one of five MG isolates in FMS (*n* = 54; Table 1). The integrity of each infection group was maintained by housing birds in separate rooms based on MG isolate. Two negative control birds that received FMS alone were present in each room. Negative control birds were never observed to have a positive eye score, confirming that the clinical signs we measured were a result of our experimental inoculation.

#### Experiment 2—western isolates

On 27 January 2011, *n* = 64 house finches from Alabama and *n* = 9 house finches from Arizona were inoculated bilaterally in the palpebral conjunctiva with 0.05 ml of FMS alone (*n* = 19) or one of five western MG isolates in FMS (Table 1). In order to account for potential host origin effects when comparing results from the two experiments, we included nine individuals from Arizona (*n* = 8 inoculated with the reference isolate [VA1994]; *n* = 1 negative control). Seven of these nine individuals (*n* = 6 inoculated with VA1994; *n* = 1 negative control) served as controls in experiment 1 (Table 1) and therefore had no prior exposure to MG. In experiment 2, VA1994 was diluted by a factor of 1∶7.37 in order to approximate the dose of CA2006. All other isolates were diluted as needed in order to match the target dose (3.04×10^6^ color changing units/ml). The integrity of each infection group was maintained by housing birds in separate rooms based on MG isolate. Two or three control birds were present in each room. In experiment 2, we used two types of control birds: (a) negative controls (*n* = 6) not previously exposed to MG, and (b) behavioral controls (*n* = 13) that were exposed to an infected cagemate during quarantine, and therefore were potentially exposed to low levels of MG. Both types of controls were eye score and quantitative PCR (qPCR) negative for MG exposure throughout experiment 2.

### Virulence

We quantified virulence, which we define as the reduction in host fitness (survival and reproduction) due to infection, using the severity and duration of inflammatory eye lesions. We used this index of virulence because prior work has shown that the presence of eye lesions is linked with higher mortality in free-living house finches [43]. In contrast, experimental infections in captivity produce little to no mortality [31],[49]. In combination, these results indicate that infection per se does not directly cause mortality, but that mortality in the wild results from visual impairment associated with the eye lesions that we measure. Disease prevalence is negligible during the breeding season [30], so direct effects of infection on reproductive rates are not important in this system. Thus, we used eye score in captivity as a relevant proxy for decreased fitness associated with disease severity (virulence) in the wild.

To score lesions, all birds were examined on day 0 PI, day 7 PI, and then weekly until day 56 PI. Lesions were scored on a 0 to 3 scale (as per [75]): 0 = no detectable swelling or eversion; 1 = minor swelling around the eye ring, 2 = moderate swelling and eversion of the conjunctival tissue, and 3 = the eye nearly hidden by swelling and crusted exudate.

### Pathogen Load

MG presence and load were quantified using qPCR as described in [76]. Each bird's eyes were individually swabbed at days 0, 7, 14, 21, 28, 42, and 56 PI. A sterile cotton swab dipped in tryptose phosphate broth was inserted into each conjunctiva. Following 5 s of swabbing in the conjunctiva, the swab was placed directly into 300 µl of sterile tryptose phosphate broth. Swabs were swirled and wrung out on the inside of the tubes to remove liquid from them before they were discarded. Samples were kept on ice and then frozen at −20°C prior to DNA extraction.

Genomic DNA was extracted from all conjunctival swabs using Qiagen DNeasy 96 Blood and Tissue kits (Qiagen). As per [76], we used primers and a probe that target a portion of the *mgc2* gene of MG that is conserved across songbird-derived MG isolates (unpublished data). Each 25-µl reaction contained 12.5 µl of iQ Supermix (2×), 0.65 µl each of 10 µM forward and reverse primers, 0.35 µl of 10 mM probe, 5.85 µl DNase-free water, and 5 µl of DNA sample. Cycling was performed using a MyiQ Single Color Real-Time PCR Detection System (Bio-Rad) and the following parameters: 95°C for 3 min, and 40 cycles of 95°C for 3 s and 60°C for 30 s, with a ramp rate at 0.5 degrees/s. Standard curves were generated for each run. The standard was based on 10-fold serial dilutions of plasmid containing a 303-bp *mgc2* insert [76]. The curve was created using 1.15×10^2^ to 1.15×10^8^ copy numbers. In experiment 1, two negative control individuals were qPCR positive in one eye on a single day PI (*n* = 1 on day 14 and *n* = 1 on day 21). Because these individuals never developed lesions, they were not positive at any other point throughout the experiment, and the detected pathogen levels were very low (log 2.24–3.08) and limited to one eye, these results were assumed to reflect sampling or assay error rather than exposure to MG.

### Statistical Analyses

We used generalized linear mixed models implemented with hierarchical Bayesian methods that accounted for bird-to-bird variation in response to infection and for correlated day-to-day changes in response through time for each combination of host origin, isolate of the pathogen, and experiment (Methods S1). All models were fit using Markov chain Monte Carlo methods implemented in WinBUGS [77]. Because of the ordinal nature of the eye score response, we used the cumulative logistic link to model eye scores relative to the asymptomatic state (eye score of zero); this can be thought of as three logistic regressions that predict the probability of a positive eye score (1, 2, or 3) as a function of host individual, pathogen isolate, and day PI. We then used these probabilities to estimate the expected (average) eye score as a function of the covariates. For pathogen load data, we assumed that the observed response (qPCR) came from a two-step process where zero observations are modeled as originating from a different process than positive observations; these models are often referred to as zero-inflated models and are commonly used for ecological count data [78].

To make the results more interpretable, we calculated derived quantities from the estimated parameters at the mean of the individual bird effects. For eye score and pathogen load, we calculated mean response values for each isolate at each day PI (Equation 4, Methods S1); we refer to these as “average eye score” and “observed pathogen load,” respectively. We quantified the pathogen component of virulence for each isolate (“average virulence index”) by additionally averaging over the eight sampled days PI (Equation 5, Methods S1). We quantified an “average pathogen load” similarly for each isolate. Finally, we calculated the correlation between an isolate's average virulence index and average pathogen load in two ways. First, we calculated the correlation by removing the average of the isolates within an experiment/isolate origin (east or west). This provided a minimum, conservative correlation between virulence and pathogen load, in which the variation due both to experiment and isolate/host origin were removed. We also calculated a maximum correlation coefficient as the observed effect without removing the mean of isolate/host origin.

We examined the effect of experiment and host origin on virulence only for VA1994 because this isolate was used in both experiments and was therefore inoculated at different doses (experiment 1 versus 2; Table 1) and into hosts of multiple geographic origins within experiment 2 (Table 1). Because we found significant host and experimental effects for VA1994 (Figure S1), our quantitative inferences are restricted to within-host-population and within-experiment comparisons only. Qualitative between-experiment inferences are made only with respect to the reference isolate (VA1994). Statistical methods, including WinBUGS code, are explained fully in Methods S1.

## Supporting Information

Figure S1 **Host origin (Arizona or Alabama) and experiment (1 versus 2) effects on virulence (eye score) of** ***M. gallisepticum*** **.** Mean effects are plotted for each observation day PI. AL, Alabama; AZ, Arizona. Bars represent 95% Bayesian credible intervals, and are graphed only for VA1994.2.AL in order to improve clarity.
(TIFF)

Figure S2 **Expected pathogen load (log_10_ scale), given a positive observation, in the conjunctiva of house finches inoculated with** ***M. gallisepticum*** **isolates.** Isolates were eastern (A) (experiment 1) or western (B) (experiment 2) in origin, and means are presented for each observation day PI.
(TIFF)

Figure S3 **Probability of observing a positive pathogen load in the conjunctiva of house finches inoculated with** ***M. gallisepticum*** **isolates.** Isolates were eastern (A) (experiment 1) or western (B) (experiment 2) in origin, and means are presented for each observation day PI. Results are plotted on a logit scale in order to improve clarity.
(TIFF)

Figure S4 **The relationship between the mean pathogen load of four index birds used to initiate an epidemic (in two flocks per isolate) and the probability that a naïve house finch became infected 26 d after introduction of one of three** ***M. gallisepticum*** **isolates.** Bars represent standard errors around the mean for each isolate. See Methods S2 for experimental details and results.
(TIFF)

Methods S1 **Detailed statistical methods and WinBUGS model code.**
(DOCX)

Methods S2 **Methods and results for a related experiment that examined within-flock transmission rates of three** ***M. gallisepticum*** **isolates that vary in virulence.**
(DOCX)

## Abbreviations

FMS: Frey's broth medium with 15% (volume/volume) swine serum
MG: *Mycoplasma gallisepticum*
PI: post-inoculation
qPCR: quantitative PCR
