# Supplementary figures and images for: Parallel Patterns of Increased Virulence in a Recently Emerged Wildlife Pathogen
Source: PLoS Biol. 2013 May 28;11(5):e1001570. doi: 10.1371/journal.pbio.1001570 (PMC3665845; doi:10.1371/journal.pbio.1001570)

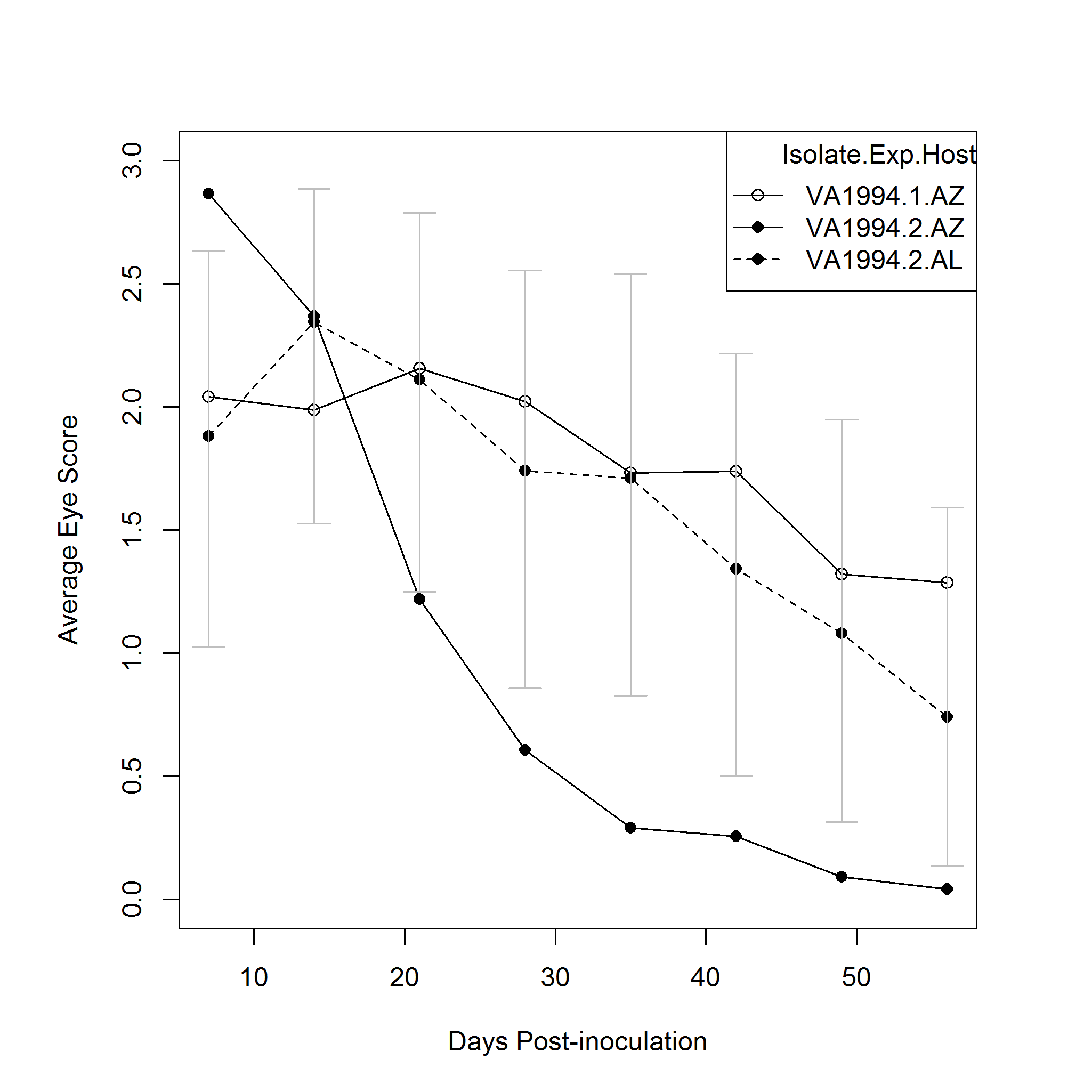

Supplement: Figure S1 — Host origin (Arizona or Alabama) and experiment (1 versus 2) effects on virulence (eye score) of M. gallisepticum . Mean effects are plotted for each observation day PI. AL, Alabama; AZ, Arizona. Bars represent 95% Bayesian credible intervals, and are graphed only for VA1994.2.AL in order to improve clarity. (TIFF) [file pbio.1001570.s001.tiff]

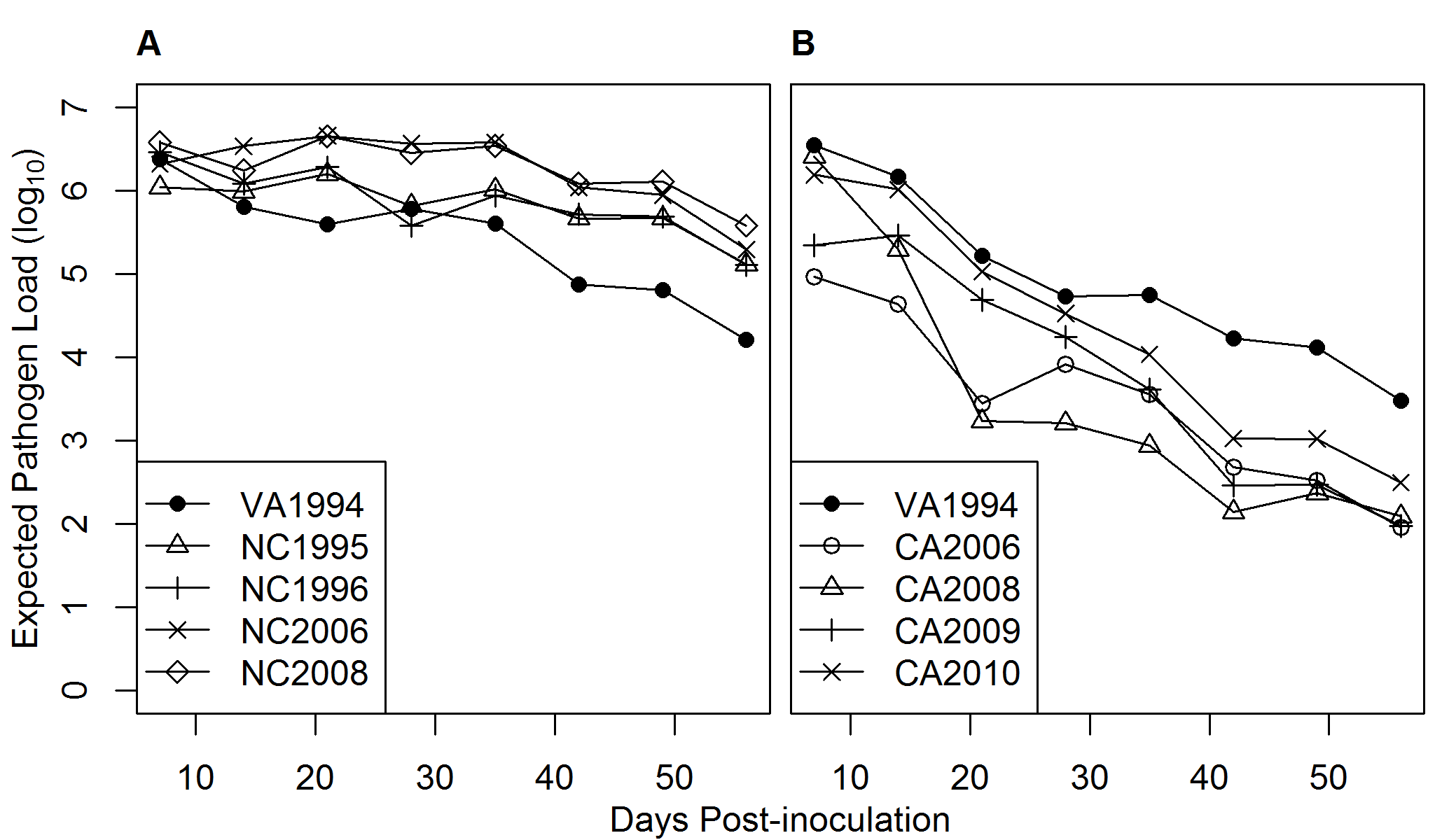

Supplement: Figure S2 — Expected pathogen load (log10 scale), given a positive observation, in the conjunctiva of house finches inoculated with M. gallisepticum isolates. Isolates were eastern (A) (experiment 1) or western (B) (experiment 2) in origin, and means are presented for each observation day PI. (TIFF) [file pbio.1001570.s002.tiff]

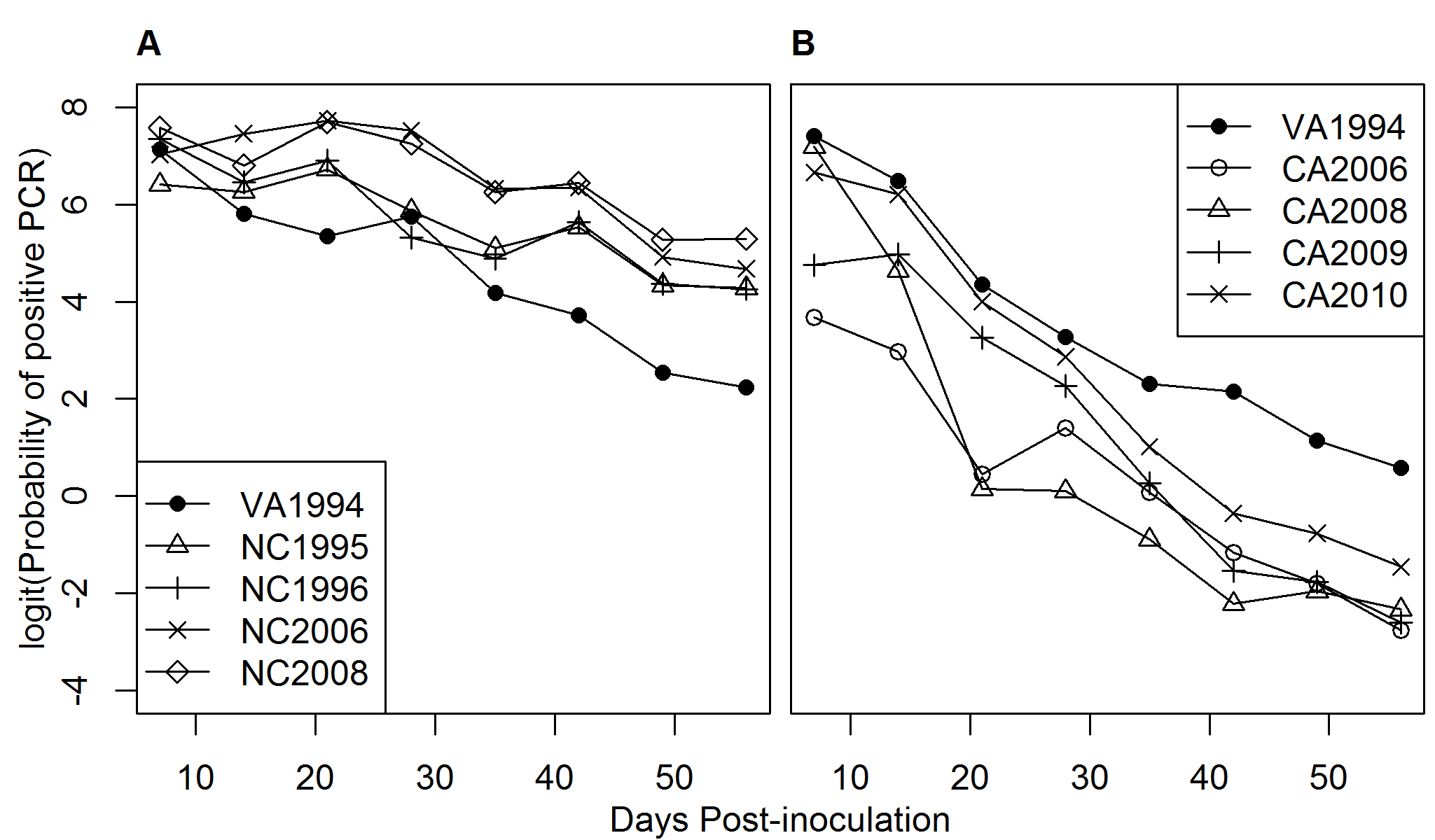

Supplement: Figure S3 — Probability of observing a positive pathogen load in the conjunctiva of house finches inoculated with M. gallisepticum isolates. Isolates were eastern (A) (experiment 1) or western (B) (experiment 2) in origin, and means are presented for each observation day PI. Results are plotted on a logit scale in order to improve clarity. (TIFF) [file pbio.1001570.s003.tiff]
