# Supplementary material for: Parallel Patterns of Increased Virulence in a Recently Emerged Wildlife Pathogen
Source: PLoS Biol. 2013 May 28;11(5):e1001570. doi: 10.1371/journal.pbio.1001570 (PMC3665845; doi:10.1371/journal.pbio.1001570)
Supplement: Methods S1 — Detailed statistical methods and WinBUGS model code. (DOCX) [file pbio.1001570.s005.docx]

**S1. Supporting Information**

*Statistical Methods*

We examined the influence of MG isolate, experiment, host population origin, and day post-inoculation on eye score and pathogen load using a hierarchical, generalized linear mixed model fit using Markov chain Monte Carlo methods in the program WinBUGS[[1](#_ENREF_1)]. Because all host populations or isolates did not occur in each experiment (Table 1), we fit separate effects for each isolate-experiment-host-day combination and allowed for serial correlation between neighboring effects in time. Below, we refer to each separate isolate-experiment-host combination as a “isolate” (although in the WinBUGS code below the terms “strain” and “inoculum” are used synonymously) and then we construct specific contrasts between estimated parameters to examine the day-specific, or average effect (over days), of experiment or host. Because of the ordinal nature of the “eye-score” response we used the cumulative logistic link to relate eye-scores to the linear predictor. For the linear predictor, we explored a variety of structures, but as in past analyses [[2](#_ENREF_2),[3](#_ENREF_3)] we found that the best models all contained a isolate-by-days post-inoculation (PI) interaction; thus, we report results based on the linear predictor:


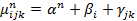
 (1)

Where,
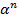

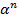
is the eye score specific intercept for the ordinal level (superscript *n* indexes the eye score levels 0, 1, and 2). An index (or linear predictor) for the highest eye score level is not needed because the probability of this level is determined by the compliment of the next lower level;
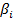

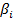
 is a random effect for individual birds indexed by *i*, and
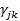

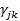
is a random effect for each isolate (all experiment-host-isolate combinations, indexed by *j*) and day PI (indexed by *k*) combination.

We used relatively uninformative prior distributions for all parameters. The prior for the intercept parameters,
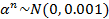

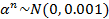
, was a normal distribution with a zero mean and precision (inverse of the variance) of 0.001. In addition the constraint
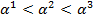

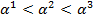
was imposed, which is necessary to model the cumulative probability of a response. The prior for the individual bird effect was
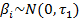

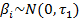
. For the isolate-specific day effects, we specified
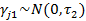

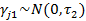
 for the first observation post-inoculation (day 7) and
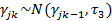

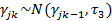
 for each subsequent observation. Thus, there is a serial autocorrelation structure to these day effects, where the precision parameter
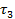

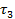
corresponds to one week; these precision parameters were then assigned a Gamma hyperprior
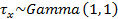

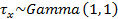
. None of the results presented here were sensitive to the choice of priors and hyperpriors.

For pathogen load data, we assume that the observed response (qPCR) came from a two-step process where zero observations are modeled as originating from a different process than positive observations. These models are often referred to as zero-inflated models and are commonly used for ecological count data [[4](#_ENREF_4)]. The first process determines whether any pathogen was observed at all. Such a process could be due to pathogen clearance by the host immune response, or due to a detection threshold effect in either the swabbing or the qPCR method. Together, these processes produce observations of zero pathogen load with probability
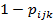

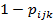
; we refer to this as the “zero process”. We model this probability using a logistic link of a linear predictor. The second process is the actual observed positive value of pathogen load, given that the first process led to a positive response; we refer to this process as the “regular process”. Here we assumed that this distribution was lognormal with an expectation given by the log-link of a linear predictor. For the linear predictor,
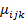

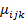
, we used an identical structure as for eye score (above) but with only one intercept term, *α^1^*, because pathogen load is a ratio-scale variable (hence the lack of superscripts). For the zero process, we use the linear predictor
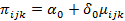

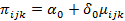
. Thus, there is a relationship between the expectation of the regular process and the probability of observing a positive response that is controlled by the parameters
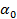

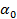
 and
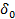

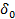
. Taken together, the likelihood of a zero pathogen load observation is
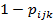

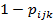
 and for a positive observation is
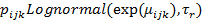

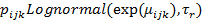
. Priors and hyperpriors for
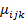

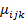
were specified identically as for the ordinal eye score model. The prior for the lognormal precision parameter was
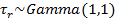

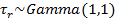
. Priors for
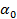

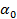
 and
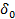

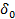
 were specified as
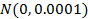

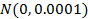
.

To make the results more interpretable, we calculated derived quantities from the model’s estimated parameters and monitored their posterior distributions. All quantities were calculated at the mean of the individual bird effects (
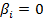

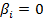
). For eye score, we calculated mean eye score for each isolate-day PI by first calculating the probability of an eye score

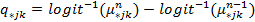
 (2)

For *n = 2,3* and as


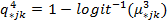
 (3)

For *n = 4* and where
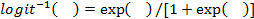

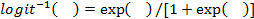
 is the inverse link function. Then mean eye score for each isolate-day combination was calculated as


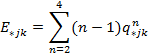
 (4)

For pathogen load, we examined the probability of a positive pathogen load observation,
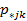

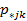
, and calculated two derived quantities and their average over the 8 observed days PI (indexed by *k*). First, we calculated the expected pathogen load given that a positive load was observed,
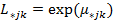

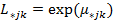
. Third, we calculated the model-predicted expectation of observed pathogen load,
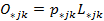

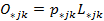
. We also calculated the average over days PI for each isolate for each quantity (
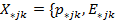

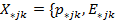
,
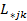

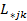
,
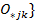

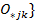
) as


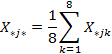
 (5)

Posterior distributions of the parameters and derived quantities were determined by initializing four chains in different locations of parameter space and then allowing a burn in for 50000 iterations per chain. We then monitored samples for 50000 more iterations per chain to determine the posterior distributions of parameters and derived quantities. Chains appeared to converge in < 10000 iterations according to the Gelman-Rubin statistic [[5](#_ENREF_5)]. We monitored and used the deviance information criterion (DIC, [[6](#_ENREF_6)]) to compare the presented model to simpler models. These simpler models included one with isolate and day effects but without an isolate-by-day interaction and another identical to the presented model but without a correlation between adjacent day effects (i.e., exchangeable day effects within an isolate).

The initial fit of the eye score and pathogen load models strongly emphasized the importance of isolate-specific autocorrelated effects for successive days (difference in posterior mean DIC scores > 100 for day-by-isolate interaction and >6 for autocorrelation). Thus, we report results based only on the models that includes these effects.

**References Cited**

1. Spiegelhalter DJ, Best, N.G., Carlin, B.R., van der Linde, A. (2004) WinBugs user manual. Version 1.4.3. Cambridge, England: MRC Biostatistics Unit.

2. Hawley DM, Dhondt KV, Dobson AP, Grodio JL, Hochachka WM, et al. (2010) Common garden experiment reveals pathogen isolate but no host genetic diversity effect on the dynamics of an emerging wildlife disease. J Evol Biol 23: 1680-1688.

3. Grodio JL, Hawley DM, Osnas EE, Ley DH, Dhondt KV, et al. (2012) Pathogenicity and immunogenicity of three Mycoplasma gallisepticum isolates in house finches (Carpodacus mexicanus). Vet Microbiol155: 53-61.

4. Martin TG, Wintle BA, Rhodes JR, Kuhnert PM, Field SA, et al. (2005) Zero tolerance ecology: improving ecological inference by modelling the source of zero observations. Ecol Lett 8: 1235-1246.

5. Brooks SP, Gelman A (1998) General methods for monitoring convergence of iterative simulations. J Comput Graph Stat 7: 434-455.

6. Spiegelhalter DJ, Best NG, Carlin BR, van der Linde A (2002) Bayesian measures of model complexity and fit. J R Stat Soc Series B Stat Methodol 64: 583-616.

**WinBUGS model code**

Ordinal Eye Score Model

#ordinal response model for HOFI eye score, response must be 1,2,3,4

#number of observations = 843

#eye score = 1,2,3,4

#Day = day post-inoculation, 7,14,21,..., 56 -> 1, 2, 3, ..., 8

#Bird_ID = individual bird id number 1:106

#Inco = inoculum (pathogen), 1,2, ...9

#Inco2 = inoculum-experiment-host combination, see Table 1 in Manuscript, 1:11

## This model adds a Day by Inco interaction and maintains the AR1 correlation structure for all day effects

##Here there is no average day effect nor any average inoculum effect only the interaction between day and Inco

##All effect are defined as deviations between grand mean and effect (cell means model)

##Parameters: beta0[1:3] are eye score specific intercepts, alpha[] is the bird effect, delta[i,j] is the effect of inoculum j on day i.

## tau[1:3] are precision parameters

model{

#Cut points

beta0[1] ~ dnorm(0, 0.001) I(,beta0[2])

beta0[2] ~ dnorm(0, 0.001) I(beta0[1],beta0[3])

beta0[3] ~ dnorm(0, 0.001) I(beta0[2],)

#Likeihood

for(i in 1:843){

logit(Q[i,1]) <- beta0[1] + alpha[Bird_ID[i]] + delta[Day[i],Inco2[i]]

logit(Q[i,2]) <- beta0[2] + alpha[Bird_ID[i]] + delta[Day[i],Inco2[i]]

logit(Q[i,3]) <- beta0[3] + alpha[Bird_ID[i]] + delta[Day[i],Inco2[i]]

#place holder for other effects in data set

null[i] <- Inco[i] + Room[i] + Sex[i] + Exp[i] + Host[i] + Bird_ID[i] + Ran[i]

#find probabilities from cumulative probabilities

p[i,1] <- Q[i,1]

p[i,2] <- Q[i,2] - Q[i,1]

p[i,3] <- Q[i,3] - Q[i,2]

p[i,4] <- 1-Q[i,3]

for(j in 1:2){ Eye[i,j] ~ dcat(p[i,1:4]) }

}#End Likeihood

#priors

for(i in 1:106){ alpha[i] ~ dnorm(0,tau[1])}[[7](#_ENREF_7)]

for(i in 1:11){ delta[1,i] ~ dnorm(0, tau[2])}[[7](#_ENREF_7)]

for(i in 2:8){for(j in 1:11){ delta[i,j] ~ dnorm(delta[i-1,j],tau[3]) }}

#hyper priors

for(i in 1:3){ tau[i] ~ dgamma(1,1) }

##Calculate some derived quantities, other calculated outside WinBUGS

##Calculate mean eye score by day and inoculum

for(j in 1:11){

for(i in 1:8){

logit(pEye1[i,j]) <- beta0[1] + delta[i,j]

logit(pEye2[i,j]) <- beta0[2] + delta[i,j]

logit(pEye3[i,j]) <- beta0[3] + delta[i,j]

meanEye[i,j] <- 1*(pEye2[i,j]-pEye1[i,j]) + 2*(pEye3[i,j]-pEye2[i,j]) + 3*(1-pEye3[i,j])

}

meanStrain[j] <- (1/8)*sum(meanEye[,j])

}

##Strain codes: 9 is VA94 in Exp. 1 with AZ host, 10 is VA94 in Exp. 2 with AL, and 11 is VA94 in Exp.2 with AZ

##Strain codes 1:4 are CA08:CA10 all in Exp. 2 and host AL

##Strain codes 5:8 are NC95:NC06 all in Exp. 1 and host AZ

##Host codes: Al=1, AZ=2

##Experiment codes: Exp 1 = 1, Exp 2 = 2

}#End Model

Zero-inflated lognormal pathogen load Model

#zero-inflated lognormal response model for HOFI qPCR

#number of observations = < 1265, NA are missing values

# the number of 0's in R or L eye = 215

#Day = day post-inoculation, 7,14,21,..., 56 -> 1, 2, 3, 4, 6, 8 -> days 35 and 49 (codes 5,7) are missing

#Bird_ID = individual bird id number 1:106

#Inco2 = inoculum (pathogen)-host-experiment combination, 1,2, ...11

## This model includes a Day by Inco interaction and maintains the AR1 correlation structure to all day effects (model 3.3 for eye score)

##All effects are defined as deviations between grand mean and effect (cell means model)

##Here for qPCR the is a mixture distribution between a Bernoulli detection probably (p) and a lognormal distribution for number of ##genome copies

##Here p varies by bird, and day-strain combination

##Parameters for pathogen load: alpha1 is intercept, beta1 is bird effect, delta1[i,j] are effect for day i and inoculum j

##Parameters for probability of detection: alpha0[1] is the background detection (intercept) parameter, alpha0[2] controls effect of ##pathogen load on detection.

## tau[1:3] and tau_ln are precision parameters

model{

#Likeihood

#Zeros

for(i in 1:215){

#placeholder for unused data

null[i] <- side[i] + Inco[i]

Y[i] <- 0

mu[i] <- alpha1 + beta1[Bird_ID[i]] + delta1[Day[i],Inco2[i]]

logit(p[i]) <- alpha0[1] + alpha0[2]*mu[i]

Y[i] ~ dbern(p[i])

}

#nonzeros

for(i in 216:1265){

null[i] <- side[i] + Inco[i]

Y[i] <- 1

mu[i] <- alpha1 + beta1[Bird_ID[i]] + delta1[Day[i],Inco2[i]]

logit(p[i]) <- alpha0[1] + alpha0[2]*mu[i]

Y[i] ~ dbern(p[i])

qPCR[i] ~ dlnorm(mu[i],tau_ln)

}#End Likeihood

#priors

for(i in 1:2){ alpha0[i] ~ dnorm(0, 0.0001)}

alpha1 ~ dnorm(0, 0.0001)

for(i in 1:106){ beta1[i] ~ dnorm(0,tau[1])}

for(i in 1:11){ delta1[1,i] ~ dnorm(0, tau[2])}

for(i in 2:8){for(j in 1:11){ delta1[i,j] ~ dnorm(delta1[i-1,j],tau[3]) }}

tau_ln ~ dgamma(1,1)

#hyper priors

for(i in 1:3){ tau[i] ~ dgamma(1,1) }

##Calculate mean qPCR by day and inoculum

for(j in 1:11){

for(i in 1:8){

pred[i,j] <- alpha1 + delta1[i,j]

logit(pobs[i,j]) <- alpha0[1] + alpha0[2]*pred[i,j] #predicted probability of positive qPCR

log(E_PCR[i,j]) <- pred[i,j] #expected qPCR given positive qPCR

E_obs[i,j] <- pobs[i,j]*E_PCR[i,j] #expected observed qPCR

}

#Calculate above quantities by strain

meanStrain[1,j] <- (1/8)*sum(pobs[,j])

meanStrain[2,j] <- (1/8)*sum(E_PCR[,j])

meanStrain[3,j] <- (1/8)*sum(E_obs[,j])

}

}#End Model
