# Supplementary material for: Parallel Patterns of Increased Virulence in a Recently Emerged Wildlife Pathogen
Source: PLoS Biol. 2013 May 28;11(5):e1001570. doi: 10.1371/journal.pbio.1001570 (PMC3665845; doi:10.1371/journal.pbio.1001570)
Supplement: Methods S2 — Methods and results for a related experiment that examined within-flock transmission rates of three M. gallisepticum isolates that vary in virulence. (DOCX) [file pbio.1001570.s006.docx]

**S2. Supporting Information- Transmission Experiment**

**Materials and Methods**

In 6 identical octagonal aviaries (volume 17.87 m^3^ each) inside a closed barn, we introduced 12 wild-caught house finches that had been captured in Tompkins County, NY in summer and fall of 2009 and in which we had been unable to detect previous exposure to MG: all birds were negative for MG as determined by the absence of MG-specific antibodies (ELISA, as per [[1](#_ENREF_1)]), the absence of MG DNA (qPCR, as per [[2](#_ENREF_2)]) and the absence of external conjunctival signs of disease (eyescore, as per [[3](#_ENREF_3)]). On February 4, 2010 all birds were captured and baseline samples were taken to confirm the absence of MG infection (blood, conjunctival swabs). Two individuals in each group (hereafter referred to as index birds) were selected at random and inoculated in the conjunctiva with 0.5 ml of an inoculum (see below), kept in a paper bag for 10 minutes to allow complete absorption of the inoculum, and released back into their respective aviary.

In each of two aviaries we used one of three MG isolates for which virulence was characterized in the full manuscript (Table 1): CA2006 2006.052-5, no dilution; VA1994 7994-1, dilution 1:7.4; or NC2006 2006.080-5 (4p) dilution 1:100. The objective of the dilutions was to inoculate all index birds with a similar dose of MG (3.04 x 10^6^ Color changing units / mL).

On day 4 post-inoculation (PI), we scored the severity of eye lesions and took conjunctival swabs from each eye as described above. Thereafter, lesions were scored and conjuctival swabs taken weekly, but on off-set days for each aviary within treatment (days 11, 18, 25, 32, 39, 46, 58, 70 PI or days 12,19, 26, 33, 41, 47, 59, and 70). Within individuals, we summed the number of copies of MG detected via qPCR and log-transformed the total as our measure of pathogen load.

We quantified the date of transmission as the first date a bird was positive by qPCR in either eye. We calculated ‘survivorship’ of healthy birds (e.g. time to infection) using a Kaplan-Meier survivorship analysis in Statistix 8 (Analytical Software, Tallahassee FL, USA). We compared survivorship between groups using the Log-Rank test as it allows data to be censored and does not assume a particular survival function. We here report transmission as the probability that a naïve birds becomes qPCR positive by day 26 PI (1 - survival).

Although the load of MG that we introduced into each index bird was the same regardless of isolate identity, pathogen growth rates differed within the index birds across isolates (a pattern documented in Figure 4 of the full manuscript). As a measure of the amount of pathogen available in each aviary to result in initial transmission, we averaged the pathogen load of the 4 index birds per isolate over the first two weeks of infection (days 4 and 11 or 12 PI), when birds are most infectious [[4](#_ENREF_4)]). By censoring transmission data at day 26, we were able to measure transmission caused primarily by the index birds and to minimize contributions of non-index birds to transmission.

**Results**

We detected significant differences in transmission between the three isolates (Logrank test: Chi-square = 33.28; df=2; P<0.0001). Transmission was highest in cages where the NC2006 isolate was introduced, and lowest in groups where CA2006 was introduced. The differences in transmission across isolates are, at least in part, explained by differences in MG-load of the index bird (Figure S4).

**References Cited**

1. Hawley DM, Grodio J, Frasca S, Kirkpatrick L, Ley DH (2011) Experimental infection of domestic canaries (Serinus canaria domestica) with Mycoplasma gallisepticum: a new model system for a wildlife disease. Avian Pathol 40: 321-327.

2. Grodio JL, Dhondt KV, O'Connell PH, Schat KA (2008) Detection and quantification of Mycoplasma gallisepticum genome load in conjunctival samples of experimentally infected house finches (Carpodacus mexicanus) using real-time polymerase chain reaction. Avian Pathol 37: 385-391.

3. Sydenstricker KV, Dhondt AA, Ley DH, Kollias GV (2005) Re-exposure of captive house finches that recovered from Mycoplasma gallisepticum infection. J Wildl Dis 41: 326-333.

4. Dhondt AA, Dhondt KV, McCleery BV (2008) Comparative infectiousness of three passerine bird species after experimental inoculation with Mycoplasma gallisepticum. Avian Pathol 37: 635-640.
